# Tracking the development of the petaloid fertile stamen in *Canna indica*: insights into the origin of androecial petaloidy in the Zingiberales

**DOI:** 10.1093/aobpla/plt009

**Published:** 2013-02-18

**Authors:** Ana M. R. Almeida, Andrew Brown, Chelsea D. Specht

**Affiliations:** 1 Department of Plant and Microbial Biology and the University and Jepson Herbaria, University of California, Berkeley, CA 94720, USA; 2 Department of Integrative Biology, University of California, Berkeley, CA 94720, USA

**Keywords:** *Canna*, evo-devo, floral development, MADS-box genes, petaloid stamens, petaloidy, Zingiberales

## Abstract

Flowers of the order Zingiberales demonstrate a remarkable trend of reduction in the number of fertile stamens; from five or six fertile, filamentous stamens bearing two thecae each in Musaceae and Strelitziaceae to just a single petaloid stamen bearing a single theca in Cannaceae and Marantaceae. As one progresses from ancestral to derived floral forms, 5–6 fertile stamens are replaced by 4–5 petaloid staminodes. In Cannaceae and Costaceae, all members of the androecial whorls exhibit petaloidy, including the fertile stamen. In Costaceae, a single fertile stamen develops two thecae embedded on a broad petaloid appendage, while in Cannaceae the single fertile stamen is further reduced to a single theca with a prominent, expanded petaloid appendage. Whether petaloidy of the fertile stamen is a synapomorphy of the entire ginger clade (including Cannaceae, Costaceae, Zingiberaceae and Marantaceae), or the result of independent convergent evolution in Cannaceae, Costaceae, and some Zingiberaceae, is unclear. We combine a developmental series of the formation of the petaloid fertile stamen in *Canna indica* with data on the expression of B- and C-class floral organ identity genes to elucidate the organogenetic identity of the petaloid stamen and staminodes. Our data indicate that the single fertile theca in *C. indica* and its petaloid appendage are derived from one-half of the primordium of a single stamen, with no contribution from the remaining part of the stamen (i.e. the second theca primordium) which aborts early in development. The petaloid appendage expands later, and develops from the position of the filament/connective of the developing theca. Floral identity gene expression shows that petal identity genes (i.e. B-class genes) are expressed in all floral organs studied while C-class gene AG-1 is expressed in an increasing gradient from sepals to gynoecium, and AG-2 is expressed in all floral organs except the petals. The canonical model for molecular specification of floral organ identity is not sufficient to explain petaloidy in the androecial whorl in *Canna* sp. Further studies understanding the regulation of gene networks are required.

## Abstract (precis)

The order Zingiberales comprises ∼2500 species of tropical to subtropical plants, including agriculturally (e.g. banana, ginger) and horticulturally (e.g. cannas, heliconias, bird-of-paradise) important plants. Throughout the evolution of this order, the stamens have been modified from the ancestral filamentous structures that produce pollen (seen in *Banana* flowers) to petal-like structures that no longer bear pollen sacs (seen in *Canna* flowers). This results in a reduction of pollen, but an effective increase in the overall size of the floral display and perhaps in the efficacy of specialized pollinators by converting stamens into ‘petals’. This study investigates the genetic mechanisms that are involved in making petal-like structures in place of pollen-producing stamens.

## Introduction

The Zingiberales are a group of herbaceous tropical monocots comprising eight families and ∼2000 species. They diverged from their sister order Commelinales (Bremer *et al.* 2009) ∼80 million years ago. In Zingiberales, the flowers are organized into five distinct whorls of three organs each: calyx (consisting of three sepals), corolla (consisting of three petals), two androecial whorls for a total of six (three inner and three outer) stamens and the tripartite gynoecium (Kirchoff 1983).

**Figure 1.**
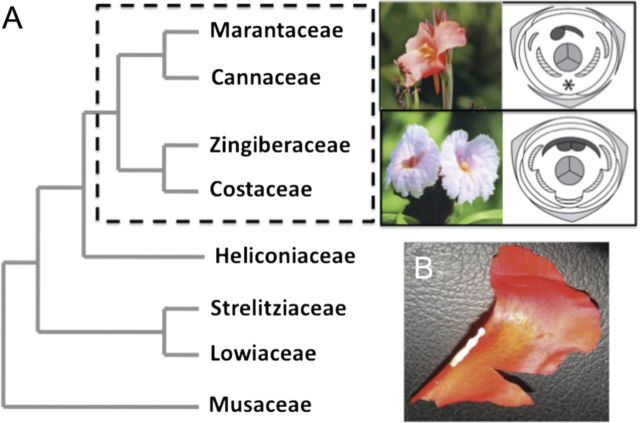
Phylogenetic context for studying comparative organogenesis in Zingiberales. (A) Zingiberales phylogeny according to molecular and morphological characteristics (Kress 1990; Kress *et al.* 2001). The dashed square highlights the ginger clade, comprising a monophyletic group of four families (Costaceae, Zingiberaceae, Cannaceae and Marantaceae). Photographs: *C. indica* (top); *Costus spicatus* (bottom). On the right, floral diagrams representative of flowers of the Cannaceae (top) and Costaceae (bottom) families. Light grey, sepals; white, petals; hashed, petaloid staminodes; dark grey, fertile stamen; *, aborted stamen; centre grey, gynoecium. (B) *Canna indica* half fertile stamen with petaloid appendage.

The Zingiberales order has been traditionally divided into two groups based on overall floral morphology: the banana families, including families Musaceae, Lowiaceae, Strelitziaceae and Heliconiaceae, and the derived ginger families, a monophyletic lineage containing families Costaceae, Zingiberaceae, Marantaceae and Cannaceae (Fig. 1A). Most major evolutionary changes in floral morphology that define these two groups occur in the petal and stamen whorls. In particular, there is an impressive reduction in the number of fertile stamens across the order, from 5–6 fertile stamens in the banana families to a single fertile stamen in Costaceae and Zingiberaceae, and a half fertile stamen in Cannaceae and Marantaceae (Kirchoff *et al.* 2009). In the flowers of the ginger families, three to five infertile members of the androecial whorls develop as sterile petaloid structures (Kirchoff 1991).

In most Zingiberales flowers, the fertile stamens produce two mature pollen sacs or thecae. In the banana families, these fertile stamens have a narrow connective and thus are filamentous in form. Any petaloid members of the androecial whorls of the banana families are infertile, completely lacking thecae (Kirchoff *et al*. 2009). However, in the ginger clade a petaloid appendage can develop from the filament or connective of the fertile members of the androecial whorl (Fig. 1B) (Kirchoff 1991; Glinos and Cocucci 2011). This results in the potential for all members of the androecial whorls, whether fertile or sterile, to develop petaloidy.

In *Costus scaber*, the anther consists of two locules, positioned adjacent to each other on the ventral surface of a petaloid structure in the inner androecial whorl (Kirchoff 1988). Development of the petaloid component of the fertile stamen, which includes both filament and connective, is simultaneous with the development of the anther (Kirchoff 1988). The stamen primordium is divided into two parts—the ventral portion produces the anthers and the dorsal portion produces the petaloid filament and connective (Kirchoff 1988). Conversely, in the Zingiberaceae (sister to Costaceae; Fig. 1A), Leinfellner characterized the petaloid component of the fertile stamen as occurring late in development, thus classifying the petaloid portion as an accessory structure (Leinfellner 1956) and implying lack of homology between the petaloid structures in the fertile stamens of Costaceae and Zingiberaceae.

The concentric androecial whorls of *Canna indica* consist of 3–4 petaloid staminodes (sterile) and one-half of a single fertile petaloid stamen (Glinos and Cocucci 2011). The fertile stamen, labellum and inner staminode constitute the inner androecial whorl, while the outer androecial whorl is made up of the two (or sometimes one) remaining staminodes (Eichler 1875; Rao and Donde 1955; Pai 1963; Kirchoff 1983). According to Kirchoff (1988, 1991), the fertile stamen is always found in the inner androecial whorl, which develops before the outer androecial whorl. However, the developmental origin of the petaloid appendage of the fertile stamen in Cannaceae remains unclear.

Our understanding of the molecular basis of floral development has increased greatly since the first descriptions of the genes responsible for specifying the identity of floral organs in *Antirrhinum* and *Arabidopsis* (Bowman *et al*. 1991; Jack *et al*. 1992). According to the canonical ABC model of floral development (Weigel and Meyerowitz 1994), differential gene expression results in the specification of the identity of the various floral organs. In *Arabidopsis*, A-class genes [*APETALA2* (*AP2*) and *APETALA1* (*AP1*)] are involved in the specification of sepals (first whorl organ), and together with B-class genes [*GLOBOSA* (*GLO*) or *PISTALLATA* (*PI*), and *DEFICIENS (DEF) or APETALA3* (*AP3*)] they specify petal identity (second whorl). B-class genes are also involved in the specification of stamen identity when expressed together with the C-class gene [*AGAMOUS* (*AG*)]. Furthermore, *AG* alone is responsible for the specification of carpel identity (Coen and Meyerowitz 1991). Although most components of the ABC model of floral development hold true for most model species studied so far, it is unclear to what extent this model can explain the morphological diversity and evolution of floral development across angiosperms. In the case of monocots, the most well-studied systems are among the grasses where the highly derived flower morphology of the Poaceae renders statements of homology a difficult task.

In *C. indica*, it is unclear whether the petaloid appendage of the half fertile stamen is produced by the secondary expansion of residual meristematic tissue from the filament of a single fertile theca, or whether it is a result of a homeotic transformation of one of the thecae into a petaloid structure. Here, we use developmental studies to characterize the origin of the petaloid tissue in the *Canna* stamen and investigate whether the combinatorial expression of MADS-box genes can explain petaloidy in *C. indica* androecial whorls.

## Methods

### Developmental series

Living material of *Canna* sp. was collected from the UC Berkeley Botanical Gardens, the Specht Lab diversity collection at the Oxford Tract Greenhouses, from residential neighbourhoods in the Berkeley hills (with consent from home-owners) and from the UC Berkeley Student Organic Garden (SOGA) (Table 1). In total, 30 inflorescences were collected from *C. indica* (18), *Canna edulis* (4), *Canna tuerckheimii* (4) and *Canna* sp. (4). Although several *Canna* species were observed in order to characterize any potential differences across Cannaceae, the developmental series portrayed and the molecular characterization focus specifically on the development of *C. indica*.

**Table 1:** Accession of *Canna* sp. used in morphological and molecular studies of floral developmental evolution.

Accession no.	Voucher location	Species	Fig. 2
AB006	SOGA	*Canna indica* L.	2H
AB009	UC Botanical Gardens	*Canna edulis* Ker Gawl.	2C
AB017	UC Botanical Gardens	*Canna indica* L.	2G
AB020	SOGA	*Canna indica* L.	2A, B, D–F, I

Inflorescences were dissected from living material, removing the outer bracts to expose most floral buds and floral organ primordia at the inflorescence apex. The apices were vacuum infiltrated for 10–20 min in formalin–acetic acid–alcohol (FAA) (3.7 % formaldehyde), and stored in cold FAA for up to 2 weeks. Tissue fixation was carried out using a standard microwave procedure (Schichnes *et al*. 1999) as follows: three rounds of 15–min microwave cycles at 37 °C, followed by an ethanol dehydration series (50, 70, 95 and 100 % ethanol) for 5 min at 67 °C for each ethanol concentration. Tissue was stained in 1 % w/v fast green FCF in 100 % ethanol for 2–3 days at 4 °C. Subsequently, tissue was destained with 100 % ethanol for 2–5 days at 4 °C, as necessary for final dissection, observation and photography (Sattler 1968).

Inflorescences were further dissected under an Olympus dissecting scope, and photographs were taken using a ×3.8 Ultrapak epi-illumination objective (Posluszny *et al*. 1980; Charlton *et al*. 1989) on a Leitz Orthoplan microscope equipped with a Nikon Digital Sight 5M digital camera, as described by Bartlett *et al.* (2008). NIS Elements software was used to process the images taken at different focal points (Bartlett *et al.* 2008) to expand the depth of focus.

### Gene expression

*Canna indica* flowers were dissected from the same plants as used above. Fresh flowers were quickly dissected, separating sepals, petals, staminodes, petaloid part of the fertile stamen, anther of the fertile stamen and gynoecium into separate vials. RNA was extracted from each of the floral parts individually. RNA extraction was carried out from fresh tissue with Plant RNA Reagent (Invitrogen), according to the manufacturer's guidelines. cDNA was synthesized after DNase treatment of each sample (Fermentas) using a BIO-RAD iScript Reverse Transcription Supermix kit with poliT primers. Reverse transcription (RT) primers were designed for *AGAMOUS-1* and *AGAMOUS-2* (*AG-1* and *AG-2*), *DEFICIENS* (*DEF*), and *GLOBOSA-1* and *GLOBOSA-2* (*GLO-1* and *GLO-2*). *GLO* sequences were downloaded from NCBI (GU594924.1 and GU594945.1) and used for RT primer design. *DEF* and *AG* genes were first amplified using degenerate primers. Polymerase chain reaction (PCR) products were cloned into Top10 cells and sequenced using an ABI Big Dye Terminator kit on a 3700 sequencer. Recovered sequences were used to develop copy-specific RT primers.

Primer sequences are as follows: *GLO1* Forward, CCC TTC CAC GTT ATC GAC GAT T; *GLO2* Forward, CGT CCA CCT CGT TGT CTG AG; *GLO* Reverse, TTG TGC ATC TTC CAA ATC TCC; *DEF* Forward, CCT CCA CTG AAA CAA AGA AGA TT; *DEF* Reverse, CAG TTC ATG CAG CAA GTT CC; *AG1* Forward, AGC CTA TGA ATT GTC GGT CTT G; *AG1* Reverse, AGC TGA GAG ACT CAC CCA TCA; *AG2* Forward, CGT ACG AAT TGT CCG TGC TT; *AG2* Reverse, TCT GCT CTC GAG TTG CTT CA. Reverse transcription-polymerase chain reactions were carried out using a Phire DNA Polymerase kit (Finnzymes) and the following: 2 μL of 5× Phire buffer; 0.2 μL of 10 mM dNTPs; 0.5 μL of each primer; 0.1 μl of Phire polymerase; 1 μL of [1:10]cDNA; and ddH_2_O, for a total volume of 10 μL. Thermocycling conditions followed the manufacturer's recommendations, and the following annealing temperatures for 30 cycles: *GLO1*, 66 °C; *GLO2*, 68 °C; *DEF*, 63 °C; *AG1* and *AG2*, 70 °C.

Reverse transcription-polymerase chain reactions were visualized on 1 % agarose gels, and stained with GelRed^TM^ (Phoenix Research Products) according to the manufacturer's protocol.

## Results

### *Canna indica* fertile stamen development

*Canna indica* early floral development has been described previously (Kirchoff 1983). Here, we present only our new developmental data focused on the fertile stamen in order to understand the origin of the petaloid appendage. Therefore, early stages of floral development are only briefly discussed.

**Figure 2.**
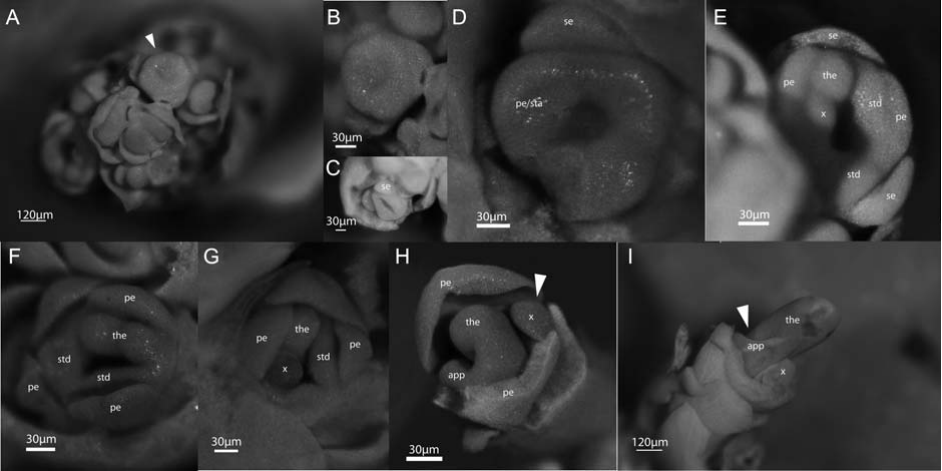
*Canna indica* floral development series. (A) Floral initiation showing the protrusion of the sepal primordial. The arrowhead points to a floral primordium amplified in (B); (B, C) development of the ‘floral cup’; (D) sepal primordia already separated from the remaining floral primordium, and evident petal primordia; (E) early stages of fertile stamen development, with two theca primordia; (F, G) fertile stamen development; (H) later stages of fertile stamen development. A single theca has developed with its petaloid appendage, while the other theca arrests development (arrowhead); (I) an almost mature stamen with its petaloid appendage (arrowhead); and the aborted theca to its right. se, sepal; pe, petal; pe/sta, petal/stamen common primordium; std, staminode; the, fertile theca; app, fertile stamen appendage; x, aborted theca primordium.

The earliest discernible stage in *C. indica* floral development (Stage 1; Fig. 2A) is represented by the development of two meristematic bulges, previously described as the sepal primordia (Kirchoff 1983). As the floral bud continues to develop, the apex flattens out, forming a disc-shaped structure, the ‘floral cup’ (Stages 2, 3; Fig. 2B). The periphery of the floral cup continues to grow and differentiate, eventually becoming delineated into the distinct petal and stamen primordia (Stages 4, 5; Fig. 2C, D). At about Stage 6 (Fig. 2E–G), the young fertile stamen protrudes out of the floral cup, distinguishing itself from the young petals. These observations are consistent with *Canna* floral development that has been well documented and described until Stage 6 (Rao and Donde 1955; Pai 1963; Kirchoff 1983).

Stages 7 and 8 (Fig. 2H, I) depict the continued growth of the fertile stamen and the determination of organ identity. By Stage 7, the rapid development of the fertile stamen and its accompanying petaloid appendage becomes evident, and becomes a distinct feature in the floral bud (Fig. 2H). One theca continues to develop, while the other becomes comparatively reduced in size and discontinues growth or expansion (Fig. 2H). The petaloid appendage is connected to the developing theca along the filament and apparently below where the connective would normally develop (Fig. 2I). Owing to the abortion of the second theca, no connective region is apparent.

At Stage 8 (Fig. 2I), the final stage of this developmental series, the nearly mature fertile stamen is represented by a single developed theca that is connected to a rapidly expanding petaloid appendage emerging from the filament. A line of cleavage separates the aborted theca from the growing fertile theca with its petaloid appendage.

### Gene expression during floral development

**Figure 3.**
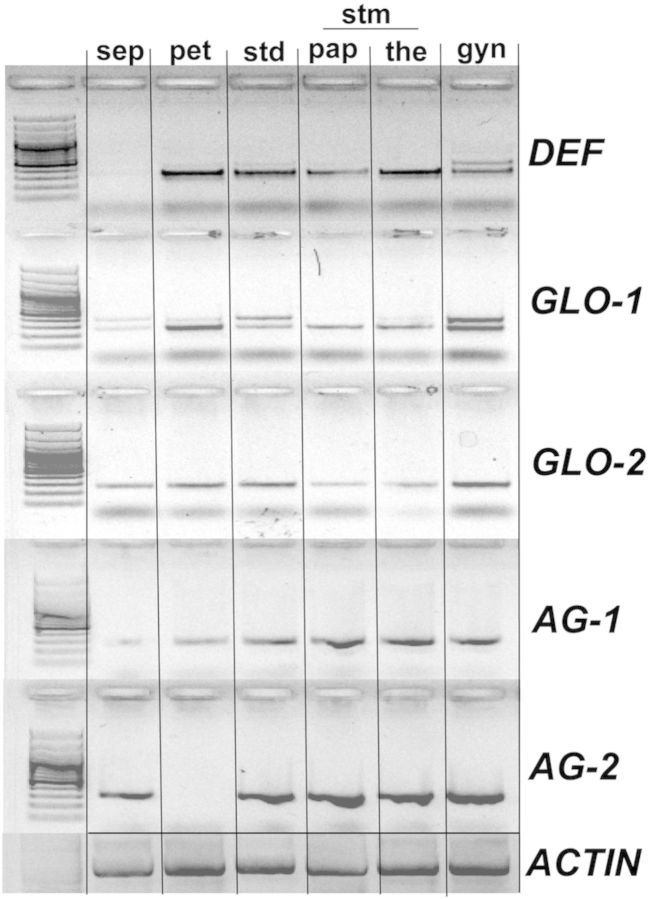
Expressions of B- and C-class MADS-box genes in the floral organs of *C. indica* as detected by RT-PCR. Each *C. indica* floral organ was dissected and RNA was extracted independently. The fertile stamen was divided into petaloid appendage and theca. sep, sepal; pet, petal; std, staminode; stm, stamen; pap, petaloid appendage of stamen; the, theca; gyn, gynoecium. Actin was used as an endogenous control for the cDNA synthesis. B-class genes: *DEF*, *DEFICIENS*; *GLO-1*, *GLOBOSA-1*; *GLO-2*, *GLOBOSA-2*. C-class genes: *AG-1*, *AGAMOUS-1*; *AG-2*, *AGAMOUS-2*.

Reverse transcription-polymerase chain reaction for *C. indica* was used in order to assess the expression pattern of B- and C-class MADS-box genes in various floral organs (Fig. 3). Sepals (sep), petals (pet), staminodes (std) and gynoecium (gyn) were studied in their entirety. For a better account of gene expression patterns on *Canna* organs, the fertile stamen was divided into petaloid appendage (pap) and theca (the), which were studied independently. *Canna indica* has at least one copy of *DEFICIENS* (*DEF*), two copies of *GLOBOSA* (herein referred to as *GLO-1* and *GLO-2*) and two copies of *AGAMOUS* (*AG-1* and *AG-2*) (A. M. R. Almeida *et al*., unpubl. data).

B-class MADS-box genes (*DEF*, *GLO-1* and *GLO-2*) are expressed in all the floral parts studied (Fig. 3). It is interesting to note that expression of these genes is reduced in sepals, especially for *DEF* and *GLO-1*. B-class gene expression shows an expanded pattern when compared with the *Arabidopsis* ABC model, where expression of the B-class genes is restricted to petals and stamens. C-class MADS-box genes (*AG-1* and *AG-2*) also show an expanded pattern of expression when compared with the expected expression pattern based on the canonical ABC model (Fig. 3): *AG-1* seems to be expressed in a gradient, increasing from sepals (low) to gynoecium (high), while *AG-2* is evenly expressed in all floral parts studied with the exception of the petals, where no expression was observed.

## Discussion

The initial stages of organogenesis in this developmental series confirm past studies on *Canna* floral development (Rao and Donde 1955; Pai 1963; Kirchoff 1983). Here we focus on the development of the fertile stamen with particular attention given to its petaloid appendage.

**Figure 4.**
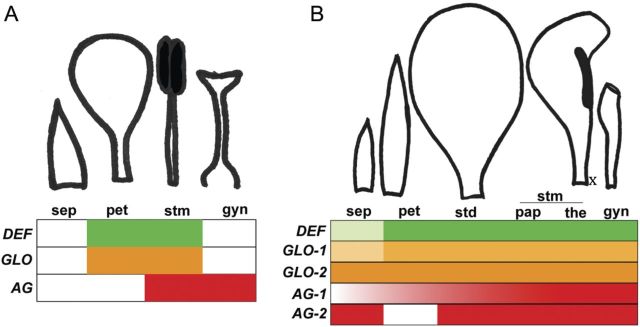
Summary results for gene expression and the corresponding floral organ morphology in *Arabidopsis* and *Canna*. (A) Classical ABC model of floral development based on *Arabidosis thaliana*. Only B-class (*DEFICIENS* and *GLOBOSA*) and C-class (*AGAMOUS*) MADS-box genes are depicted, as the role of A-class MADS-box genes in floral development in monocots awaits further investigation. In the classical ABC model, petal identity is a result of A- and B-class MADS-box gene expression, while stamen identity results from concomitant expression of B- and C-class MADS-box genes. (B) *Canna indica* B- and C-class MADS-box gene expression pattern. *Canna indica* contains two *GLOBOSA* genes (*GLO-1* and *GLO-2*) and two *AGAMOUS* genes (*AG-1* and *AG-2*). B- and C-class MADS-box genes are expressed in most of the floral parts studied here, and when compared with the classical ABC model, show an expansion in their expression domains. x, position of the aborted theca primordium relative to the half fertile stamen.

Petaloidy is a striking trend in the evolution of Zingiberales floral morphology, especially in the ginger clade where the number of fertile stamens is drastically reduced and the remaining infertile androecial members are petaloid. The extreme case is observed in Cannaceae flowers, in which all androecial elements are petaloid and the half fertile stamen has a marked petaloid appendage (Fig. 1B). In this case, only one theca is apparent at anthesis, and the question remains whether (i) the petaloid appendage of the fertile stamen develops from the filament and connective of the same primordium that gives rise to the single theca, or (ii) the appendage is the result of the growth and expansion of a separate theca primordium that undergoes homeotic transformation into a sterile, petaloid structure. In the first case, only half of the original stamen primordium would develop fully, forming both the anther and the petaloid appendage (see Fig. 4B, x). In the second case, the entire stamen primordium would grow and mature, with one-half giving rise to a petaloid structure and the other half forming an anther.

The morphological series presented here (Fig. 2G–I) provides evidence for the first hypothesis: that the petaloid appendage of the *Canna* fertile stamen develops from the same primordium that produces the theca, emerging from the position of the filament. This finding has implications for understanding fertile stamen development in other genera within the ginger lineage. For instance, because it appears that the entire structure (theca and petaloid appendage) is produced from a single half (stamen) primordium, other fertile stamen configurations, such as those observed in Costaceae and Zingiberaceae, could very probably result from concerted laminar development of the filament and connective associated with both fertile thecae.

In order to investigate the molecular mechanisms associated with androecial petaloidy in *C. indica*, the expressions of class B and classC MADS-box genes were analysed in various floral organs. We did not investigate A-class gene expression, as the role of the A function genes outside of *Arabidopsis* is unclear; alternatively, B and C function has been shown to predict the stamen and petal development model for several groups of monocots (Kim *et al.* 2006; Tang *et al.* 2007). The canonical expression pattern for B- and C-class MADS-box genes (Fig. 4A) does not appear to hold for *Canna*. We expected to find B-class genes in the petal and stamen whorls, and C-class genes in stamen and gynoecium whorls, with perhaps some changes in expression defining the differences between petaloid vs. fertile stamens within the androecial whorls. Instead, B-class (*GLO* and *DEF*) genes have expression domains that are expanded in both directions to include the first whorl and the gynoecium. C-class (*AG*) genes also show a broad expression pattern and are found in petals (*AG-1*) and sepals (*AG-2*) as well as the androecial and gynoecium whorls (Fig. 4B). There was no differentiation between fertile or sterile elements within the androecial whorl, nor was there a combination that seemed to define petaloidy regardless of its whorl of origin.

As petaloidy in *C. indica* is, however, not restricted to the corolla (petal) and androecial (stamen) whorls, the extension of B-class gene expression into the gynoecium might explain the laminar morphology of the carpels in *Canna* (Glinos and Cocucci 2011). Most of the *Canna* flower shows simultaneous B- and C-class MADS-box gene expression, which in the classical ABC model would result in the specification of stamen identity. Clearly, this combination is not functioning as stamen identity in the *Canna* flower, with its single half fertile stamen. This expression pattern implies that *Canna* petaloidy, whether in the petals, stamens or even the carpels, is probably not a simple result of re-deployment of the classical petal specification mechanisms (A- and B-class MADS-box gene expression), and potentially involves an as yet uncharacterized molecular basis.

Considering the origins of stamens from a petal-like organ (Goethe 1790), it is possible that the filamentous stamens that are ancestral to Zingiberales and that characterize *Musa* flowers are the result of a restriction of laminar growth associated with the development of fertility. The lack of pollen sac production in the majority of androecial members of the ginger families might cause a de-repression of laminar growth, resulting in the production of petaloid organs in the androecial whorls. When petaloidy is found in organs that do contain fertile thecae, it is unclear as to the mechanisms that enable laminar growth in the presence of pollen sac production. Current studies are focusing on the role of polarity genes that establish the abaxial/adaxial boundary and regulate laminar vs. radial morphology of lateral organs.

## Conclusions

It is possible to conclude, based on the data from this study, that the developmental mechanisms resulting in petaloid floral organs are different even in closely related taxa such as Cannaceae and Costaceae. It appears that the development of a petaloid appendage on the filament of a single theca in *C. indica* might be the result of ectopic development resulting in the appearance of a half fertile, petaloid stamen. In contrast, in Costaceae the petaloid stamen might be the result of laminar growth of the filament and connective, returning to an ancestral leaf-like laminar development as seen in the petaloid stamens of early diverging angiosperms (e.g. *Nymphaea*). Investigations on candidate gene expression during development of the stamens in Costaceae and Cannaceae will be necessary to determine if the genetic mechanisms underlying the development of the petaloid stamens are indeed different in these two families, indicating that homoplasy can be at work even in closely related species.

## Accession Numbers

Novel sequences have been submitted to GenBank (http://www.psc.edu/general/software/packages/genbank/genbank.php) and will be released upon publication. Accession numbers: *Canna GLO1*, GU594924.1; *Canna GLO2*, GU594945.1; *Canna AGAMOUS1*, JQ180191; *Canna AGAMOUS2*, KC763343; *Canna DEF*, KC763344.

## Sources of Funding

Funding was provided by a UC Berkeley College of Natural Resources Sponsored Projects for Undergraduate Research (SPUR) student-initiated research award to A.B., a Fulbright/CAPES award to A.M.R.A. and NSF CAREER IOS 0845641 to C.D.S.

## Contributions by the Authors

All authors contributed to the writing and editing of the manuscript. Dissections and microscopy were performed by A.B. following training and mentoring by A.M.R.A. Images were interpreted and edited by A.M.R.A. and C.D.S.

## Conflict of Interest Statement

None declared.
